# Macro–Mesoscale Equivalent Evaluation of Interlayer Shear Behavior in Asphalt Pavements with a Granular Base

**DOI:** 10.3390/ma18173935

**Published:** 2025-08-22

**Authors:** Fang Wang, Zhouqi Zhang, Chaoliang Fu, Zhiping Ma

**Affiliations:** 1 School of Civil Engineering, Anhui Jianzhu University, Hefei 230601, China; fangwang@ahjzu.edu.cn (F.W.); zhangzhouqi@stu.ahjzu.edu.cn (Z.Z.); 080053@ahjzu.edu.cn (Z.M.); 2 Institute of Highway Engineering, RWTH Aachen University, 52074 Aachen, Germany

**Keywords:** asphalt pavement, gravel base, interlaminar shear strength, finite element simulation, direct shear test

## Abstract

To reduce reflective cracking in asphalt pavements, gravel base layers are commonly employed to disperse stress and delay structural damage. However, the loose nature of gravel bases results in complex interlayer contact conditions, typically involving interlocking between gravel particles in the base and aggregates in the asphalt surface course. In order to accurately simulate this interaction and to improve the interlayer shear performance, a mesoscale finite element model was developed and combined with macroscopic tests. Effects due to the type and amount of binder material, type of asphalt surface layer, and external loading on shear strength were systematically analyzed. The results indicate that SBS (Styrene–Butadiene–Styrene)-modified asphalt provides the highest interlayer strength, followed by SBR (Styrene–Butadiene Rubber)-modified emulsified asphalt and unmodified base bitumen. SBS (Styrene–Butadiene–Styrene)-modified asphalt achieves optimal interlaminar shear strength at a coating rate of 0.9 L/m^2^. Additionally, shear strength increases with applied load but decreases with increasing void ratio and the nominal maximum aggregate size of the surface course in the analyzed spectra. Based on simulation and experimental data, an equivalent macro–meso predictive model relating shear strength to key influencing factors was established. This model effectively bridges mesoscale mechanisms and practical engineering applications, providing theoretical support for the design and performance optimization of asphalt pavements with gravel bases.

## 1. Introduction

Asphalt pavements with semi-rigid base layers have been widely adopted due to their high structural strength, early-stage stability, and excellent load-bearing capacity, particularly in high-grade highways and heavy-traffic roads [1]. However, the inherent rigidity of semi-rigid bases makes them susceptible to shrinkage and fatigue cracking under the combined effects of temperature fluctuations, moisture, and repeated traffic loading [2]. These cracks often propagate upward through reflective cracking, leading to premature surface failure and reduced pavement durability. To address this issue, as a representative flexible base, granular base layers offer superior flexibility and stress-dissipating capacity, which help to interrupt crack propagation from the base to the surface. Compared with semi-rigid bases, granular bases are easier to construct and provide improved resistance to cracking, making them more suitable for asphalt pavement systems with stringent crack-control requirements [3,4,5,6,7,8].

In experimental research studies on granular bases, attention has primarily focused on material composition, structural performance, construction techniques, and their influence on asphalt pavement responses. Xia F. (2020) conducted a systematic investigation into the mechanical properties of graded crushed stone bases [9], revealing that moisture content, compaction degree, and stress conditions are critical factors affecting their dynamic modulus and deformation behavior. They found that optimizing aggregate gradation and structural parameters can significantly enhance base stiffness and resistance to permanent deformation while effectively reducing stress transmission to the surface layer, thereby helping to delay the onset of reflective cracking. Stolle et al. (2009) showed that adding reclaimed asphalt pavement aggregate to natural aggregates slightly reduced shear strength and increased deformation [10], but with proper blending and compaction, the mechanical performance could be comparable to conventional granular materials. Kazmee et al. (2017) investigated the application of RAP (Reclaimed Asphalt Pavement) in granular base layers through full-scale accelerated pavement testing [11]. The results showed that 100% RAP bases, despite having higher compaction and moduli, were prone to severe rutting under traffic loads. A 60:40 blend of recycled concrete aggregate and RAP demonstrated significantly improved performance and was identified as a more stable and sustainable granular base alternative. Xiao et al. (2011) evaluated the influence of unbound aggregate quality on flexible pavement performance [12]. They found that locally available, even marginal-quality materials can be cost-effective for low-volume roads if the traffic does not exceed 1.5 million ESALs (Equivalent Single-Axle Load). Notably, high-quality granular subbases can exhibit a bridging effect, improving subgrade protection and offsetting the rutting caused by low base stiffness.

In a numerical simulation of granular bases, E. Papadopoulos and J.C. Santamarina (2016) explored the contact behavior, stress paths, and particle rearrangements within granular bases, highlighting their influence on overall stiffness and permanent deformation under cyclic loading [13]. They found that under cyclic loading, inter-particle sliding and structural reorganization notably affected interlayer stability and shear performance. Wang H. and Al-Qadi I.L. (2012) showed that accurate simulation of the granular base requires a nonlinear anisotropic 3D model, as its modulus is affected by load, structure, asphalt viscoelasticity, temperature, and speed [14]. Ignoring its stress-dependent anisotropy may lead to large errors in fatigue and rutting predictions. Kola R. (2019) developed a rutting prediction model for flexible airfield pavements by combining the D-P-Cap-Creep model with the PANDA framework [15]. It effectively simulates permanent deformation in granular layers, including time- and rate-dependent behavior in moist, clay-rich subgrades. J.S. Shan et al. (2022) [6] concluded that a graded gravel layer can effectively mitigate reflective cracking in semi-rigid base asphalt pavements by using the discrete element method. This layer disrupts crack propagation paths, reduces stress concentration, and lowers the number of meso-level cracks. The thicker the gravel layer, the more significant the crack suppression effect, highlighting its structural benefit in controlling reflective cracking. S.D.T.E. Silva et al. (2020) [7] developed a nonlinear interface model to simulate the shear behavior between an asphalt surface and granular base layers. Based on the Mohr–Coulomb criterion and Goodman’s law, the model was validated through experiments and crack propagation simulations, demonstrating strong predictive capability for the interface response.

In summary, although significant progress has been made in experimental and numerical investigations, current research on asphalt pavements with granular bases still faces three major limitations. First, the interlayer is often oversimplified as a uniformly bonded interface, neglecting the complex aggregate embedment behavior at the contact zone. This limits our understanding of actual interlayer interaction mechanisms. Second, the combined effects of key factors—such as binder thickness, aggregate morphology, and void ratio—on interlayer shear resistance remain poorly quantified [16]. Third, there is a lack of effective integration between micromechanical simulations and macro-scale experimental data, which restricts the development of reliable predictive models for engineering applications. To overcome these challenges, this study proposes a macro–mesoscale equivalent evaluation framework for predicting interlayer shear performance in asphalt pavements with granular bases. A three-layer finite element model is established, comprising a viscoelastic asphalt surface course layer, a cohesive interlayer bonding layer, and a randomly generated granular base. Different interlocking states are simulated, while parallel macro-scale shear tests on custom non-demolding specimens quantify the effects of key design parameters, including the type and thickness of the interlayer bonding material, aggregate skeleton types, air void content, the nominal maximum size of the aggregate in the asphalt surface course, and the external load. By linking micromechanical and macroscopic behaviors, a shear-strength prediction model is developed, bridging simulation and practice to support more accurate performance assessment and pavement design.

## 2. Establishment of the Finite Element Model

To investigate the interlayer shear performance of asphalt pavement with a granular base, this study establishes a mesoscale model incorporating embedded aggregates to simulate the shear response of the composite pavement structure [17]. The entire modeling process is divided into three modules: asphalt surface course modeling, interlayer structure modeling, and granular base modeling. The detailed modeling process is described as follows.

### 2.1. Viscoelastic Modeling of the Asphalt Surface Course

Asphalt mixture is a typical viscoelastic composite material. According to rheological theory, the mechanical behavior of viscoelastic materials can be represented using elastic elements (such as springs) and viscous elements (such as dashpots), which follow Hooke’s law and Newton’s law, respectively. By combining these elements in series or parallel configurations, various classical viscoelastic models can be constructed, including the Maxwell model, Kelvin model, and Burgers model. The Maxwell model effectively describes stress relaxation; however, it fails to simulate the delayed elastic deformation in creep, while the Kelvin model captures creep but lacks stress-relaxation capability [18,19]. In contrast, the Burgers model integrates the strengths of both, enabling the simultaneous simulation of stress relaxation and creep, making it the most suitable choice for modeling the viscoelastic behavior of asphalt mixtures in this study.

In finite element analysis, the Burgers model is often implemented through a Prony series to describe the time-dependent response of materials. The shear-relaxation modulus G(t) and bulk-relaxation modulus K(t) are expressed as

(1) $$G(t)=G_{0}\sum_{i=1}^{n_{G}}{\bar{g}}_{i}exp(-\frac{t}{\tau_{i}^{G}})$$

(2) $$K(t)=K_{0}\sum_{i=1}^{n_{K}}{\bar{k}}_{i}exp(-\frac{t}{\tau_{i}^{K}})$$

*G*(*t*) is the compression relaxation (Pa) and *K*(*t*) is the volume relaxation (Pa). $G_{0}$ is the initial shear modulus and $K_{0}$ is the initial bulk modulus; these are obtained from the elastic-constant relationship between the material’s elastic modulus *E* and Poisson’s ratio *μ*. *E* and *μ* are obtained by uniaxial tensile tests. This paper selects *μ* = 0.25 [20]. The letter *n* refers to the number of units, ${\bar{g}}_{i}=G_{i}/G_{0}$ (${\bar{g}}_{i}$: shear-relaxation parameter), ${\bar{k}}_{i}=K_{i}/K_{0}$ (${\bar{k}}_{i}$: volume-relaxation parameter), and $\tau_{i}^{G}={\tau_{i}^{k}=\tau }_{\dot{i}}$ ($\tau_{\dot{i}}:$ relaxation-time parameter). In order to make the relaxation-time parameter able to fit better within one decade of relaxation, the Prony series term is selected [21,22]. The range for the relaxation-time parameter is 0.1~1.0 s. In this way, the Prony series shear-relaxation parameter ${\bar{g}}_{i}$, volume-relaxation parameter ${\bar{k}}_{i}$, and time-relaxation parameter $\tau_{\dot{i}}$ required by Abaqus can be obtained.

Among these, the initial shear modulus $G_{0}$ and the bulk modulus $K_{0}$ can be derived from the elastic modulus E (Pa) and Poisson’s ratio μ as follows:

(3) $$G_{0}=\frac{E}{2(1+\mu )}$$

(4) $$K_{0}=\frac{E}{3(1-2\mu )}$$

To comprehensively evaluate the mechanical response of different asphalt mixtures, four common mix types—AC-13, AC-16, AC-20, and AC-25—are selected in this study. Their corresponding Prony parameters are listed in Table 1 [23]. These parameters are then input into the ABAQUS platform, enabling accurate simulation of the viscoelastic response of the asphalt surface course under actual loading conditions and providing a reliable foundation for subsequent interlayer shear performance analyses.

To account for the temperature dependence of the viscoelastic behavior of asphalt mixtures, the Time–Temperature Superposition Principle is introduced, where the shift factor ($a_{T}$) is used to convert the mechanical response at one temperature to another. For linear viscoelastic materials like asphalt mixtures, the shift factor is calculated using the Williams–Landel–Ferry equation:

(5) $$log(a_{T})=\frac{-C_{1}(T-T_{0})}{C_{2}+(T-T_{0})}$$

where *T* is the target temperature (°C), *T*_0_ is the reference temperature (set to 25 °C in this study), and *C*_1_ and *C*_2_ are material constants. For 70# neat bitumen, the conventional values from asphalt material research are adopted: *C*_1_ = 16.7 and *C*_2_ = 52.4. All simulations in this paper are conducted at the reference temperature, and the results do not involve temperature variable analysis. By correcting the relaxation time ($\tau_{i}$) in the Prony series using the shift factor ($\tau_{i}^{\prime }=\tau_{i}\cdot a_{T}$), viscoelastic parameters such as the shear-relaxation modulus and bulk-relaxation modulus in Equations (1) and (2) can be extended to different temperature conditions. This correction enhances the model’s ability to reflect the time–temperature equivalence characteristics of asphalt mixtures, providing a more rigorous theoretical basis for simulating viscoelastic responses under varying temperature environments [24,25].

Additionally, for geometric modeling, a 2D rectangular domain of 150 mm × 50 mm is established to represent the asphalt surface course, following the dimensions of the composite rutting test specimen. Randomly embedded polygonal aggregates are introduced into this region to simulate the mesoscale structural heterogeneity.

### 2.2. Cohesive Modeling for the Interlayer Bonding Layer

This section presents a finite element modeling framework based on a cohesive-zone model (CZM) to simulate the interfacial behavior between the asphalt surface course and the granular base in asphalt pavements. The interlayer serves not only as a bonding medium but also as a critical load-transfer path [26]. Structural degradation such as delamination or sliding failure at the interlayer can significantly compromise the mechanical integrity of the pavement system. Therefore, developing a model that incorporates both bonding failure and aggregate interlock is essential for analyzing interlayer shear performance.

The CZM is implemented in ABAQUS using cohesive elements to represent the initiation and propagation of interface damage under shear and normal loading. Four key parameters are defined: interfacial stiffness $K_{0}^{t}$, initial maximum shear stress $\tau_{max}$, initial maximum normal stress $\sigma_{max}$, and fracture energy $\phi_{t}$. These parameters are calibrated by fitting simulated traction–separation curves, capturing the nonlinear response of the bonding material under load.

Geometrically, a 5 mm thick interlayer is modeled, reflecting the typical thickness of tack coat materials used in actual pavement construction. To account for the aggregate interlocking effect between adjacent layers, quadrilateral-shaped aggregates are embedded within the interlayer [27]. Three representative contact conditions are considered: (1) aggregates from the base partially embedded into the interlayer without contacting the surface course; (2) aggregates penetrating the full interlayer and intersecting both the upper and lower layers; and (3) aggregates from the surface that are partially embedded without reaching the base. Through CAD-based trimming and nesting operations, a realistic interlocked aggregate structure is established and exported in IGES format for simulation in ABAQUS.

In terms of material properties, three types of materials were used as the bonding material: SBS-modified 70# asphalt, SBR-modified emulsified asphalt, and neat bitumen. Coarse and fine aggregates were distinguished by size and assigned corresponding thermal and mechanical parameters. The model parameters and material parameters are listed in Table 2 and Table 3.

In summary, the developed cohesive interlayer model captures not only the damage evolution of the bonding interface but also the mechanical influence of embedded aggregates on shear resistance. It provides a physically representative and numerically robust basis for investigating interlayer instability in composite pavement structures.

### 2.3. Random Aggregate Modeling for the Granular Base

The granular base layer in pavement structures is primarily composed of crushed stone and gravel compacted to form a stable load-bearing system. Its mechanical strength depends largely on the internal frictional resistance formed through interlocking among aggregates, which is governed by the particle size distribution, surface texture, and angularity. This study focuses on the influence of embedded surface aggregates in the granular base on the interlayer shear behavior rather than on detailed modeling of the internal state of the entire base. Therefore, a 2D random-aggregate model in a loose-flow state was adopted to simulate the upper surface of the granular base. Referring to the standard dimensions of composite double-layer rutting slabs used in laboratory tests, the model size was set to 150 mm in length and 50 mm in thickness, which balances computational efficiency and physical representativeness.

The granular base is considered a composite of coarse aggregates that serve as the skeletal structure and fine aggregates that fill the interstices. A MATLAB script (MATLAB R2021a; The MathWorks, Inc., Natick, MA, USA) was used to control the generation of randomly distributed aggregate positions within the defined model boundary, based on input parameters such as the particle size range, void ratio, and the number of aggregates. The algorithm ensures that the particles do not overlap or exceed boundary limits. Once the coordinates were obtained, polygonal aggregates were drawn in AutoCAD 2021 (Autodesk, Inc., San Rafael, CA, USA) by entering the generated data, and a rectangular sketch with dimensions of 150 mm × 50 mm was established to define the model domain [28,29]. This sketch was then imported into ABAQUS to complete the initial model construction. In terms of material attributes, the base layer was designed with a 16% void ratio, in accordance with the *Specifications for Rock Tests in Highway Engineering* (JTG E41-2005). The required number of particles for each size category is summarized in Table 4.

The modeled granular base consists of coarse aggregates embedded within a matrix of finer particles, forming a quasi-homogeneous structure. Separate sections were assigned to the coarse and fine aggregates in ABAQUS to reflect their different material behavior.

The developed random-aggregate model provides a structural basis for simulating the shear interaction between the granular base and the asphalt overlay. It captures the realistic morphology of aggregate interlock and serves as a reliable reference for the finite element simulation of interlayer mechanical behavior under shear loading.

### 2.4. Finite Element Model of Asphalt Pavement with a Granular Base

**Figure 1:**
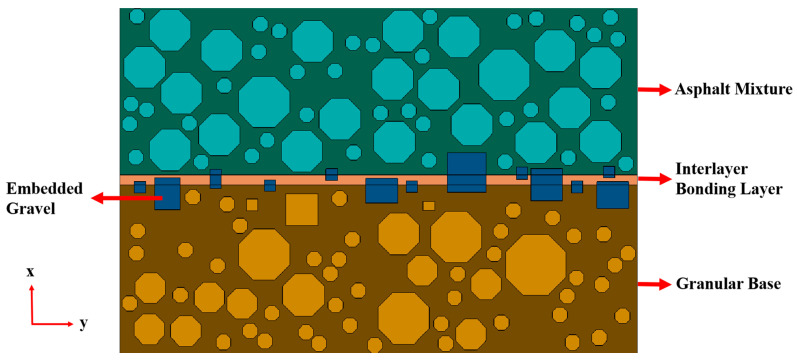
Finite element model of the composite structure model of asphalt pavement with a granular base.

To investigate the effect of aggregate interlocking on interlayer shear performance, a composite structure model of asphalt pavement with a granular base was established using the ABAQUS finite element platform. As shown in Figure 1, this model consists of three components: the granular base, the interlayer bonding layer, and the asphalt mixture surface layer. The surface layer adopts a viscoelastic material model embedded with coarse aggregates; the interlayer structure simulates the mutual interlocking of coarse aggregates protruding from both the base and the surface within the bonding material; and the base is modeled as a loose granular structure, reflecting the actual physical characteristics of the pavement.

In terms of material properties, parameters such as density, elastic modulus, Poisson’s ratio, and thermal conductivity were defined for a coarse aggregate, fine aggregate, and asphalt mixture. Sections were created and assigned to each material in ABAQUS, and the three structural parts were assembled to complete the construction of the full 2D composite model. For load and boundary-condition settings, actual traffic loading during road service was considered. The vertical tire load was simplified into a uniformly distributed load, while horizontal shear force induced by vehicle movement was the focus of the study. The x- and y-direction displacement of the asphalt surface was fixed. The direction displacement of the granular base was also fixed, and it moves horizontally in the x-direction. A constant horizontal velocity (10 mm/min) was applied to one side of the granular base to simulate vehicle-induced shear and to calculate the corresponding interlayer shear stress. The model was meshed using structured grid division. Since the interlayer structure was the key research focus, the embedded aggregates were simplified into regular quadrilaterals to improve meshing quality and computational efficiency while preserving essential geometric features. The surface and base layers were meshed structurally, and the interlayer region was meshed according to the aggregate layout. After mesh generation and model checks, the simulation was executed in ABAQUS. The resulting shear-stress cloud diagram illustrated stress concentrations at the interface, providing a foundation for evaluating the interlayer shear strength of the composite structure.

## 3. Raw Materials and the Interlayer Shear Test

### 3.1. Raw Materials

#### 3.1.1. Asphalt Mixture Surface Course

In this study, to investigate the effects of air void contents and nominal maximum aggregate size on interlayer shear strength, two batches of asphalt mixtures were prepared. First, four pavement types with different air void contents (two AC-16 mixtures with different void ratios, one AM-16 mixture, and one OGFC-16 mixture) were selected to investigate the macroscopic interlayer shear performance under varying void conditions. Among them, the air void contents of the two AC-16 mixtures were 3.4% and 5.2%, while those of AM-16 and OGFC-16 were 10.5% and 18.6%, respectively [30]. Then, four types of asphalt mixtures—AC-13, AC-16, AC-20, and AC-25—were selected to explore the effect of nominal maximum aggregate size on interlayer shear strength. All asphalt mixtures used 70# road petroleum asphalt as the binder, and their gradations are shown in Table 5.

#### 3.1.2. Interlayer Bonding Layer and Granular Base Layer

To investigate the influence of different bonding materials on interlayer shear performance, three types of materials were selected: SBS-modified 70# asphalt, SBR-modified emulsified asphalt, and neat bitumen. A preliminary evaluation of their fundamental physical properties—penetration, softening point, and ductility—was conducted, and the test results are summarized in Table 6.

The granular base layer utilized a continuously graded crushed basalt aggregate ranging from 0.075 mm to 31.5 mm, with limestone powder (particle size < 0.075 mm) as the mineral filler. The gradation composition of the granular base is shown in Table 7.

### 3.2. Sample Preparation

**Figure 2:**
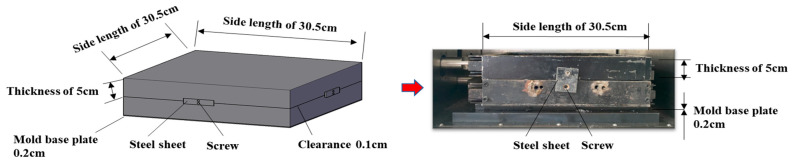
Custom-designed composite mold for interlayer shear test specimens.

The interlayer shear test specimens were prepared as double-layer composite slabs measuring 300 mm × 300 mm × 100 mm, consisting of an asphalt mixture surface layer, an interlayer bonding layer, and a granular base. Meanwhile, experimental shear tests under identical structural and loading conditions (0.7 MPa) confirmed the simulation. The preparation process strictly followed the Chinese specifications detailed in JTG D50-2017 and JTG F20-2015. To ensure structural integrity and boundary consistency during testing, all specimens were fabricated using a custom-designed steel mold. As shown in Figure 2, the mold consists of two detachable parts representing the surface and base layers, respectively. The upper mold frame is assembled from four identical steel plates (each 0.5 cm thick) screwed together to form a cavity measuring 30.5 cm × 30.5 cm × 5 cm. The lower mold is constructed from four identical side steel plates and a base plate (0.2 cm thick), also forming a cavity of the same size. Both compartments have internal dimensions of 30 cm × 30 cm × 5 cm to ensure compatibility. A controlled gap is reserved between the two structural layers to accommodate the interlayer bonding material. Steel connectors are positioned at all four sides of the mold to fasten the upper and lower components together during compaction. These connectors are removed before the direct shear test is performed, and their thickness is not considered in the structural dimensions. This specially designed mold enables precise simulation of the interlayer behavior in flexible pavement composite structures without requiring demolding, thereby preserving specimen integrity and test accuracy.

To prepare the composite specimens for interlayer shear testing, a custom-designed double-layer rutting slab mold was used to construct the granular base. According to the gradation requirements specified in the *Specifications for Design of Highway Asphalt Pavements* (JTG D50-2017), crushed aggregates of various sizes were mixed with mineral filler and blended. The mixture was then placed into the lower part of the mold and compacted using a vibration compaction device (vibration force: 6500 N; frequency: 30 Hz) to achieve the target dry density of 2.4 g/cm^3^, as recommended by the *Test Methods of Materials Stabilized with Inorganic Binders for Highway Engineering* (JTG E51-2009). After compaction, the base was cooled to room temperature to ensure structural stability. Subsequently, the interlayer bonding material—SBS-modified 70# petroleum asphalt—was heated in a high-temperature oven to its application temperature of 180 °C, ensuring that it was fully liquefied and homogeneous. It was then sprayed evenly onto the surface of the compacted granular base at an application rate of 0.9 L/m^2^. After spraying, the specimen was left to rest at ambient temperature for 24 h to allow the asphalt to gradually integrate with the base layer, enabling the protruding aggregates to embed effectively into the bonding layer and form a stable interlayer structure. Next, the pre-mixed asphalt mixture was placed into the upper part of the mold in layers and compacted using a small trowel and rubber mallet [31]. Once the upper and lower layers were assembled, the composite specimen was compacted using a rolling compactor—initially with two passes back and forth, followed by a 90-degree rotation and an additional 12 passes—to ensure strong interlayer bonding. The final composite specimen measured 300 mm × 300 mm × 100 mm and comprised the granular base, interlayer bonding layer, and dense asphalt mixture surface course, effectively simulating the interlayer performance and shear behavior of real pavement structures.

### 3.3. Interlayer Shear Test

**Figure 3:**
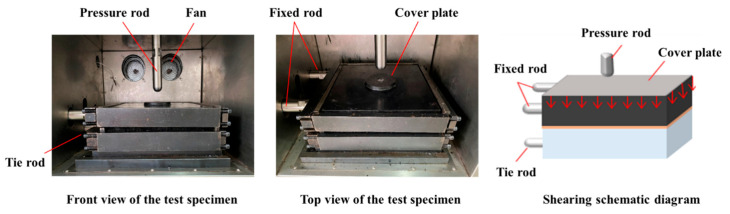
Schematic diagram of the interlayer shear test.

A large-scale direct shear apparatus was used to apply vertical shear loads. For the interlayer shear test, the specimens were placed into the shear testing apparatus, and the lateral steel frames were removed to fix the specimen position securely. In the setup, the upper pressure plate distributed a uniform vertical load through a cover plate, the tension rod applied a horizontal displacement to simulate shear action, and internal fans were used to regulate the test temperature, as shown in Figure 3. Key parameters such as vertical load, shear rate, and temperature were preset before testing.

To explore the influence of various factors on interlayer performance, five macro-level variables were considered: (1) bonding material type, (2) application rate, (3) surface mixture air voids, (4) nominal maximum aggregate size, and (5) external loading. For each factor, four levels were selected, and three replicate specimens were prepared at each level to ensure reliability. In terms of bonding materials, three types were selected: SBS-modified 70# asphalt, SBR-modified emulsified asphalt, and neat bitumen. Their key physical properties—including penetration, softening point, and ductility—were measured to evaluate their impact on shear strength [32]. Based on the pre-experiment results, the optimal application rate for SBS, SBR, and base bitumen was 0.9 L/m^2^.

Meanwhile, to examine the effect of void ratio on shear performance, four types of asphalt mixtures with varying air voids were prepared: two dense-graded AC-16 mixtures (3.4% and 5.2%), one semi-open AM-16 (10.5%), and one open-graded OGFC-16 (18.6%). For the effect of aggregate size, AC-13, AC-16, AC-20, and AC-25 mixtures were prepared, each with a different nominal maximum aggregate size but with a consistent design void ratio. Additionally, external load levels of 0, 0.3, 0.5, 0.7, and 1.1 MPa were used to simulate different traffic conditions. To isolate the effect of each individual factor on interlayer shear performance, other conditions were held constant during testing. Specifically, a uniform external load of 0.7 MPa was applied, SBS-modified 70# asphalt was used as the bonding material at its optimal application rate, and the AC-16 asphalt mixture was selected for the surface course.

The interlayer shear strength τ was calculated using Equation (6):

(6) $$\tau =\frac{Q}{A}$$

where Q is the peak interlayer shear force (*N*), and A is the effective shear area (90,000 mm^2^), based on the internal area of the slab mold.

## 4. Results and Discussion

### 4.1. Effect of the Type of Interlayer Bonding Material

**Figure 4:**
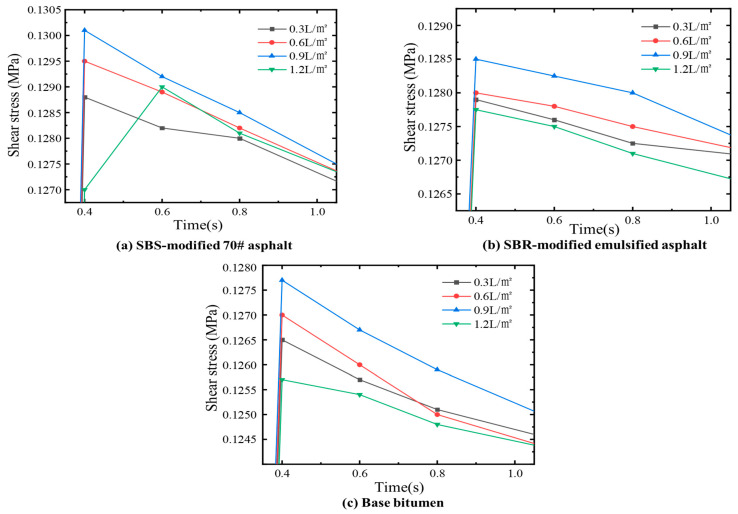
Interlayer shear strength of different types of bonding materials at various application rates.

**Figure 5:**
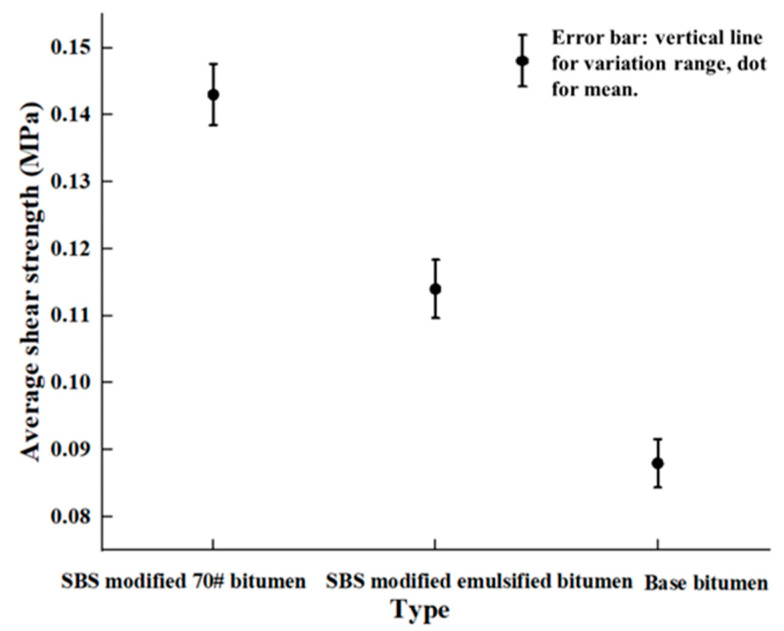
Experimental interlayer shear strength for various bonding materials.

To evaluate the interlayer bonding performance in asphalt pavement, both mesoscale simulations and macro-scale shear tests were conducted using three representative bonding materials: SBS-modified 70# asphalt, SBR-modified emulsified asphalt, and conventional base bitumen. In the mesoscale modeling framework, the spreading amount of each bonding material was equivalently converted into interlayer thickness, enabling consistent interlayer shear simulations. A series of interlayer shear tests was conducted under consistent structural and loading conditions. SBS-modified 70# asphalt, SBR-modified emulsified asphalt, and conventional base bitumen were applied at a spreading rate of 0.9 L/m^2^. As shown in Figure 4, the simulation results revealed that SBS-modified asphalt demonstrated the highest peak shear strength, followed by SBR asphalt and base bitumen. Meanwhile, experimental shear tests under identical structural and loading conditions (0.7 MPa) confirmed the simulated ranking (Figure 5 shows the average value of the maximum shear stress tested for the three groups of specimens during the shearing process). SBS-modified asphalt exhibited the strongest bonding, forming a durable interface due to its excellent adhesion and high-temperature stability, while SBR asphalt and base bitumen showed relatively poor shear resistance.

**Figure 6:**
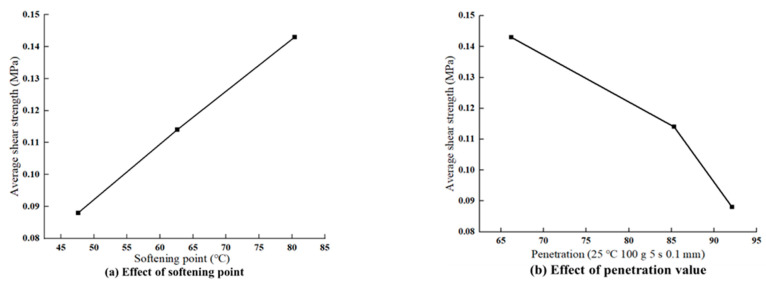
Shear strength corresponding to the softening point and penetration value of the bonding materials.

This trend correlates well with the physical and rheological properties of the materials (Figure 6) from the perspective of the softening point, which serves as a critical indicator of a binder’s thermal stability. Materials with higher softening points are more resistant to flow at elevated temperatures, which is crucial for maintaining interlayer bonding under thermal and mechanical loads. The softening point values follow the order SBS > SBR > base asphalt, which, again, aligns with the shear strength ranking. The superior softening point of SBS contributes to its ability to preserve mechanical interlock and resist adhesive softening under high-temperature shear conditions. In addition to the softening point, penetration reflects the asphalt softness and viscosity; in this aspect, the materials follow the order base > SBR > SBS—exactly inverse to the observed interlayer shear strength. A higher penetration value corresponds to a softer, less viscous material that lacks sufficient stiffness to resist shear deformation, resulting in weak bonding and a higher risk of interlayer failure. In contrast, SBS-modified asphalt, which has the lowest penetration value, exhibits greater rigidity and structural stability, enabling it to effectively maintain interface cohesion under stress. Regarding ductility, which characterizes the binder’s elongation and plastic deformation capacity, the differences among the three materials are relatively small. Given the rapid loading and stress concentration during shear testing, ductility appears to exert a minimal influence under the current experimental conditions.

In summary, both simulation and experimental results consistently indicate that interlayer bonding performance is influenced by the type of adhesive, primarily through key physical properties such as penetration and softening point. For applications requiring durable and shear-resistant pavement interlayers, it is strongly recommended to use high-performance adhesives such as SBS-modified asphalt, which combines low penetration, a high softening point, and strong bonding strength.

### 4.2. Effect of Thickness and Application Rate of the Interlayer Bonding Material

**Figure 7:**
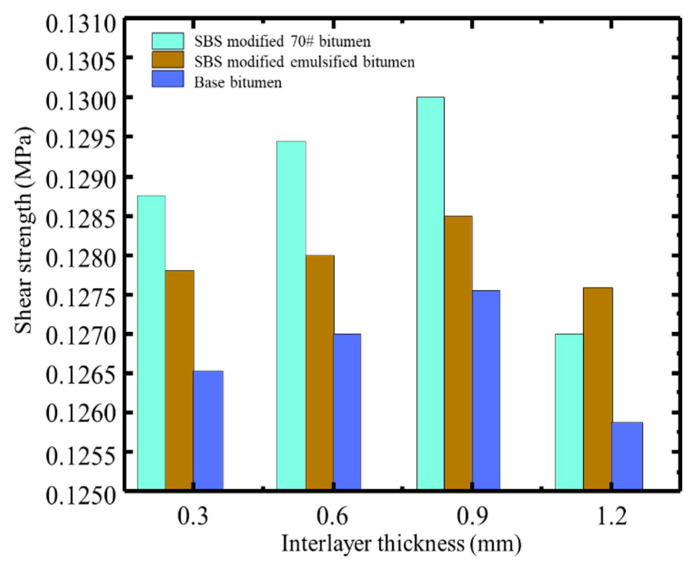
Interlayer shear strength of each bonding material at different interlayer thicknesses.

As shown in Figure 7, mesoscale simulations revealed a strong correlation between thickness and shear performance, with sensitivity varying among materials. Taking SBS-modified 70# asphalt as an example, when the interlayer thickness is 0.3 mm, only a small portion of aggregates from the surface and base layers embed into the bonding layer. In this case, the interlayer shear strength mainly relies on the frictional resistance generated by the interlocking of protruding aggregates from both layers, while the adhesive contribution of the bonding material is minimal, resulting in relatively low shear strength. As the thickness increases to 0.6 mm, in addition to a few large aggregates forming interlocking structures, the bonding material begins to provide noticeable adhesion to the embedded aggregates. The combined effect of friction and adhesion leads to a gradual increase in shear strength. When the thickness reaches 0.9 mm, aggregates from both layers can fully penetrate the bonding layer and embed into each other’s structure, and most aggregate surfaces are surrounded by the bonding material. At this point, the adhesive force of the bonding material reaches its maximum. With both frictional interlock and adhesion contributing, the interlayer structure becomes optimal, and the shear strength peaks.

In contrast, SBR-modified emulsified asphalt exhibits a relatively flat trend in shear strength, with only a slight increase at 0.6 mm. This suggests a weaker adhesive capacity and lower sensitivity to thickness changes, making it less effective in enhancing aggregate interlock. Conventional base bitumen consistently demonstrates the lowest shear strength across all thickness levels. Although a slight increase is observed at 0.9 mm, the overall improvement is limited, indicating insufficient bonding performance to generate effective aggregate interlock or adhesion.

**Figure 8:**
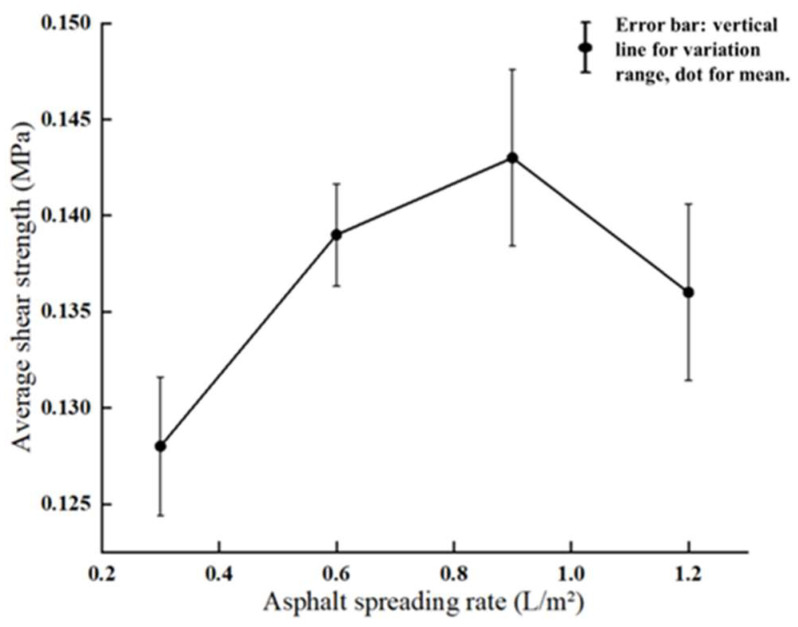
Experimental shear strength at different bonding material application rates.

Moreover, four different spreading rates—0.3 L/m^2^, 0.6 L/m^2^, 0.9 L/m^2^, and 1.2 L/m^2^—were selected for the macro-scale shear tests. The bonding material used in all cases was SBS-modified 70# asphalt. As shown in Figure 8, this exhibited a similar increase–peak–decrease pattern, with maximum shear strength observed at 0.9 L/m^2^. This trend can be explained by the mechanical behavior of the interlayer structure. When the application rate is too low, the bonding material cannot form a continuous and effective structural asphalt layer to connect the surface and base courses. As a result, the bonding strength is insufficient, and the interlayer cannot achieve strong adhesion, leading to low shear strength. With increasing application rate, the bonding material gradually covers the surface of both layers more completely, forming a continuous film of structural asphalt that significantly enhances adhesive strength and interlayer stability, thereby increasing the shear strength. However, once the application rate exceeds the optimal value, excess asphalt accumulates in the interlayer, leading to the formation of free asphalt. In this case, the adhesive force diminishes as the volume of free asphalt increases, and the interface between the surface and base layers begins to separate. Moreover, the lubricating effect of the excess asphalt reduces the frictional resistance between the protruding aggregates of the surface and base layers. As a result, the interlayer shear strength begins to decline. When the amount of free asphalt becomes excessive, the lubricating effect surpasses any adhesive benefit, ultimately causing slippage and failure of the interlayer structure.

### 4.3. Effect of the Air Void of the Asphalt Surface Course

**Figure 9:**
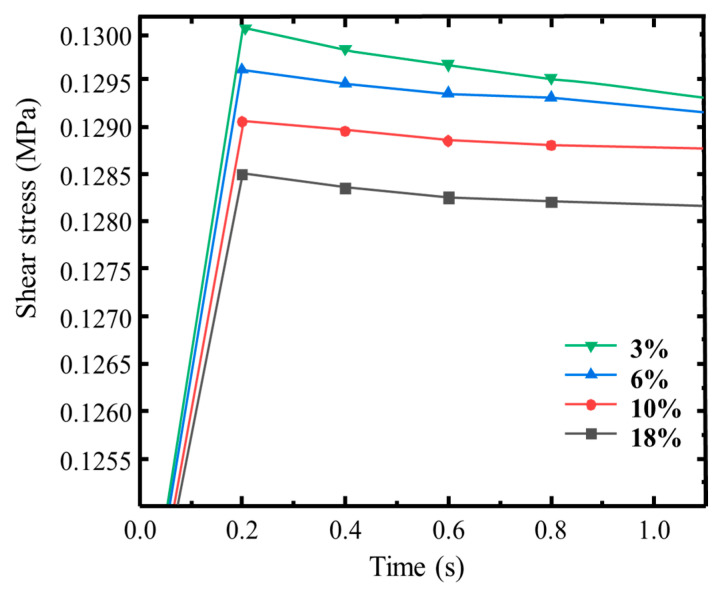
Interlayer shear strength of asphalt mixtures with different surface course air void ratios.

Four levels of air void content—3%, 6%, 10%, and 18%—were considered in the numerical simulation. As shown in Figure 9, as the air void content increases, the interlayer shear strength decreases. When the void ratio is 3%, the asphalt mixture presents the highest interlayer shear strength. At this level, the surface course has greater contact area with the interlayer, and more coarse aggregates protrude from the bottom of the surface mixture, embedding effectively into the interlayer structure. This significantly enhances internal friction. Furthermore, these aggregates also interlock more effectively with the upward-protruding stones of the granular base, forming a stable mechanical interlock. Compared with the 6% void content, the 3% mixture is more uniform and forms a more continuous contact surface with the interlayer, enabling it to resist higher levels of horizontal shear stress. At a void ratio of 10%, the shear strength decreases relative to the 3% mixture. This is attributed to the increase in structural depth and porosity, which causes the lower aggregates of the surface course to become more loosely packed, reducing their ability to effectively interlock with the base layer. Additionally, the bonding layer material fails to sufficiently wrap the protruding aggregates, further weakening the shear resistance. When the surface course has an air void content of 18%, the shear strength is the lowest among all tested configurations. The high porosity leads to a poor contact interface between the surface mixture and the interlayer. The lack of sufficient interlocking with base layer aggregates and the reduced contact area with the bonding material result in insufficient mechanical interlocking and inadequate adhesive bonding. Consequently, both internal friction and adhesion are significantly diminished, leading to the weakest interlayer shear performance.

**Figure 10:**
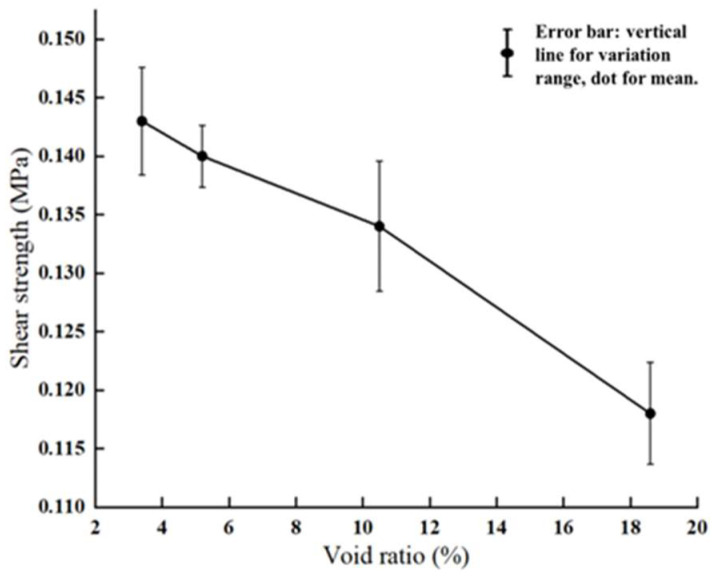
Experimental shear strength of asphalt mixture surface courses with different air void contents.

Meanwhile, surface mixtures with different air void contents—3.4%, 5.2%, 10.5%, and 18.6%—were selected in the macro-scale tests. As illustrated in Figure 10, interlayer shear strength decreases progressively as the air void content of the asphalt mixture increases. When the air void ratio is relatively low (e.g., 3.4% and 5.2%), the asphalt binder effectively fills the spaces between aggregates, improving the adhesion and interface stability between the binder and aggregate. This results in higher interlayer shear strength. In contrast, as the air void content increases to 10.5% and 18.6%, the compaction quality deteriorates, significantly weakening the binder–aggregate adhesion and thus reducing the interlayer resistance to shear. This trend is further supported by failure mode observations from post-test shear specimen interfaces. Specimens with lower air void content (3.4%, 5.2%) exhibited shear failure surfaces involving fractured aggregates, indicating that strong bonding and mechanical interlocking between layers shifted the failure to occur within the aggregate structure itself. Conversely, specimens with higher air void content (10.5%, 18.6%) showed failure surfaces dominated by debonding in the asphalt mastic, suggesting that adhesive failure at the binder–aggregate interface was the main cause of interlayer shear failure.

### 4.4. Effect of the Nominal Maximum Aggregate Size in the Asphalt Surface Course

**Figure 11:**
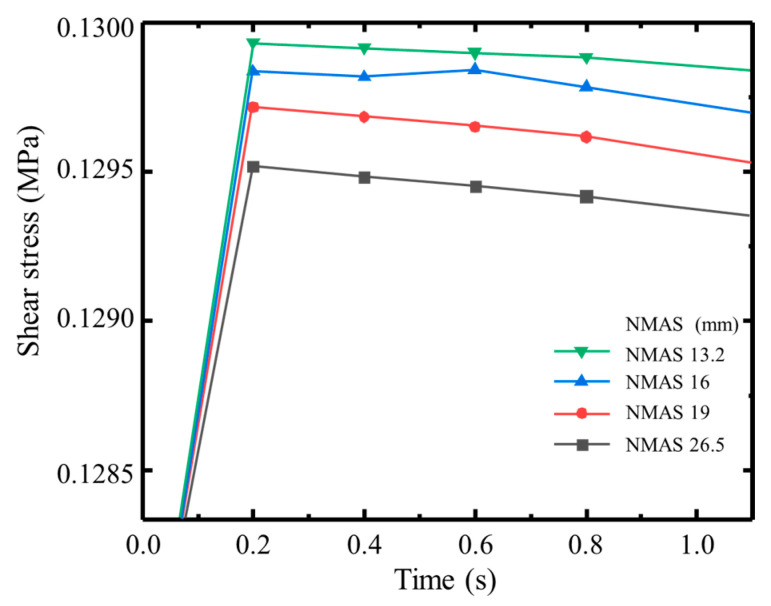
Interlayer shear strength of asphalt mixtures with different nominal maximum aggregate sizes.

**Figure 12:**
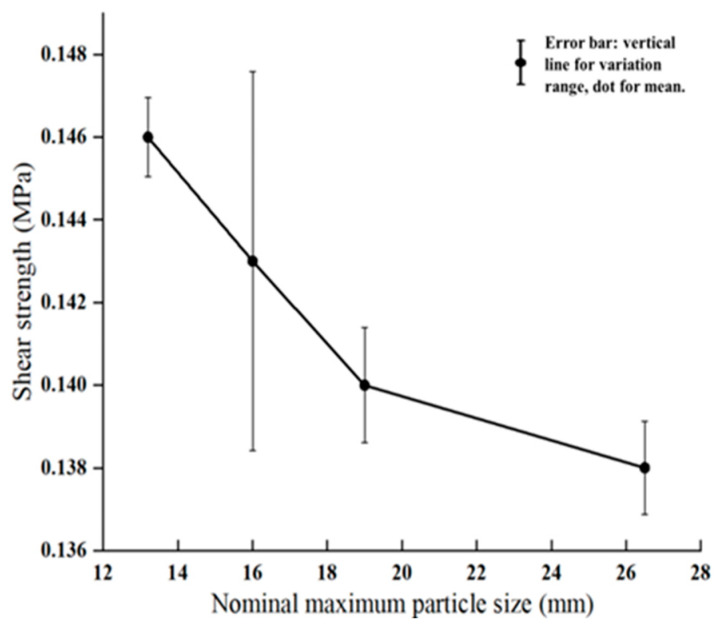
Experimental shear strength of asphalt mixtures with different nominal maximum aggregate sizes.

To investigate the effect of nominal maximum aggregate size (NMAS) on interlayer shear strength in asphalt mixtures, several mixture models were established with identical air void and asphalt binder contents but differing in their NMAS. Interlayer shear simulations were conducted, and the results are shown in Figure 11. The results clearly indicate that as the nominal maximum aggregate size increases, the interlayer shear strength significantly decreases. This trend is also confirmed by macro-scale shear tests, which showed similar results to the simulations (Figure 12). The observed phenomenon can be explained by the structural characteristics of the mixtures. A larger NMAS leads to greater spacing between coarse aggregates and larger voids, which weakens aggregate interlock and reduces internal friction and structural compactness—ultimately lowering shear resistance. In contrast, mixtures with smaller NMASs have more closely packed aggregates and smaller, more frequent voids, facilitating stronger interlocking, improved structural stability, and higher shear strength. Additionally, smaller aggregates provide a greater surface area for contact with the asphalt binder, improving adhesive bonding at the interface and further enhancing interlayer shear performance. Therefore, under otherwise identical conditions, reducing the nominal maximum aggregate size of the surface course is an effective strategy for improving interlayer bonding and structural integrity.

### 4.5. Effect of External Load

**Figure 13:**
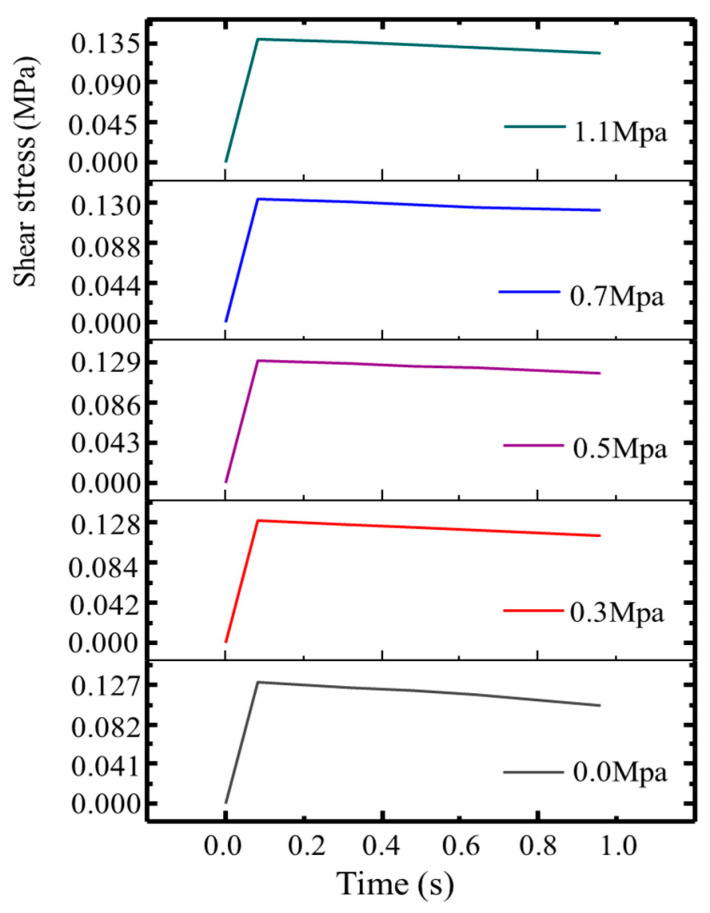
Shear strength under different external loads.

Figure 13 presents the time–shear stress curves of the pavement interlayer under various external loads (0.0 MPa, 0.3 MPa, 0.5 MPa, 0.7 MPa, and 1.1 MPa) obtained from mesoscale simulations. A consistent trend is observed: interlayer shear strength increases progressively with a higher external load. At each load level, the shear stress rises sharply at the beginning, reaches a peak, and then slightly declines or plateaus, indicating the onset of softening or partial failure under sustained loading. Under 0 MPa (no confinement), the shear strength is the lowest, as it mainly relies on initial aggregate interlocking and weak adhesive bonding. As the load increases, stress is more effectively transferred to the interlayer, enhancing both aggregate friction and adhesive contact, thereby improving the overall shear resistance. The strengthening effect is particularly noticeable between 0.3 MPa and 1.1 MPa, although excessive loading may eventually lead to structural deterioration.

**Figure 14:**
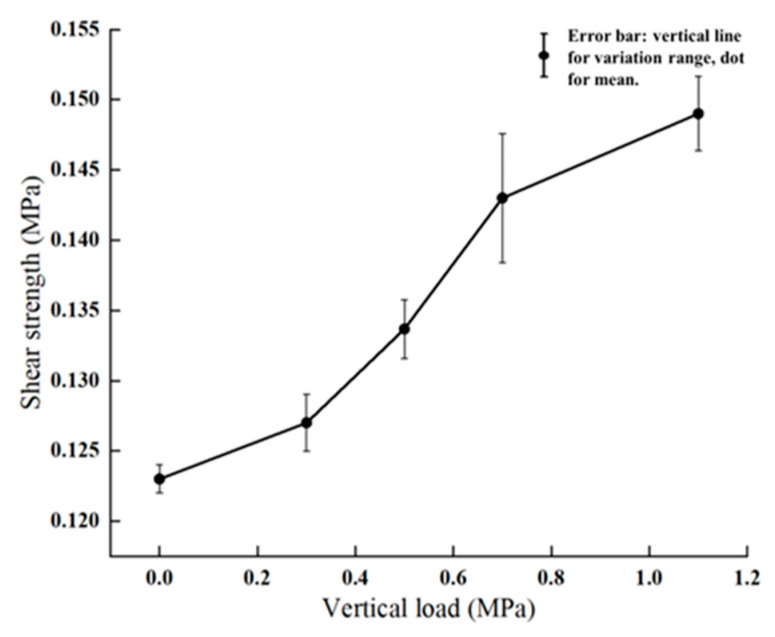
Experimental shear strength under different external loads.

Figure 14 shows the corresponding results from macro-scale shear tests, where interlayer shear strength was measured under the same range of external loads. All specimens used a consistent structure: AC-16 asphalt mixture for the surface layer and SBS-modified 70# asphalt as the bonding layer at an application rate of 0.9 L/m^2^. While the experiments only capture peak shear strength rather than the full time-dependent evolution seen in the simulation, they confirm the same increasing trend. The agreement between the simulation and test results validates the reliability of the modeling and emphasizes the practical relevance of the findings.

Both sets of results point to the same underlying mechanism: increasing vertical load enhances the mechanical interlock between surface and base aggregates and compresses the tack coat layer, strengthening adhesive bonding. This dual improvement in friction and cohesion leads to increased interlayer shear capacity. However, excessive or long-term overloading may eventually cause interface degradation or failure. Thus, in pavement design and maintenance, it is essential to control traffic-induced stress levels to ensure long-term interlayer performance and durability.

## 5. Development of a Prediction Model of Macro–Mesoscale Equivalent Interlayer Shear Strength

### 5.1. Prediction Model of Interlayer Shear Strength Based on Mesoscale Simulation

In order to quantitatively evaluate the interlayer shear performance of granular-base asphalt pavements, a prediction model was established in this study based on the results of mesoscale numerical simulation, focusing on the relationship between the interlayer shear strength (τ) and the main influencing factors, as shown in Table 8. From the table, it can be seen that the influence of different parameters on the shear strength presents different relationships. The thickness of the adhesive layer shows an obvious quadratic relationship with the shear strength: with an increase in thickness, the strength firstly increases and then decreases. This indicates that an overly thin bonding layer results in insufficient adhesion, while an excessively thick layer may cause structural slippage—implying the existence of an optimal thickness range. Similarly, the maximum nominal aggregate particle size shows a non-linear effect: the larger the aggregate particle size, the weaker the interlocking effect at the interface, which leads to a gradual decrease in shear strength. In contrast, the linear relationship between air gap content, external loading and shear strength is relatively stable. Specifically, an increase in air void content leads to a decrease in shear strength, which reflects the loosening of the material within the internal structure. On the other hand, higher external loads significantly increase the shear strength, suggesting that the loads promote the activation of interfacial bonding. From the fitting results, the coefficients of determination (R^2^) of the four variables all exceed 0.89, and some even exceed 0.95, indicating that the fitting accuracy is good.

Since the variation in interlayer shear strength is influenced by multiple factors rather than a single variable, it is necessary to develop a multi-factor prediction model to better understand its governing mechanisms. To this end, a functional relationship between the shear strength $\tau_{SIM}$ and these parameters was established through nonlinear regression analysis. We performed nonlinear fitting on the obtained experimental data using IBM SPSS Statistics version 26.0 (IBM Corp., Armonk, NY, USA) and analyzed it with a polynomial regression model. The resulting model is shown in Equation (7):

(7) $$\tau_{SIM}=-0.446+0.144h-0.226h^{2}+0.105h^{3}+0.003v+0.089d-0.005d^{2}+0.037p-\;0.098p^{2}+0.069p^{3},\;R^{2}=0.893$$

where h is the interlayer material thickness (mm); v is the air void content (%); d is the maximum nominal aggregate size (mm); and p is the external load (MPa).

**Figure 15:**
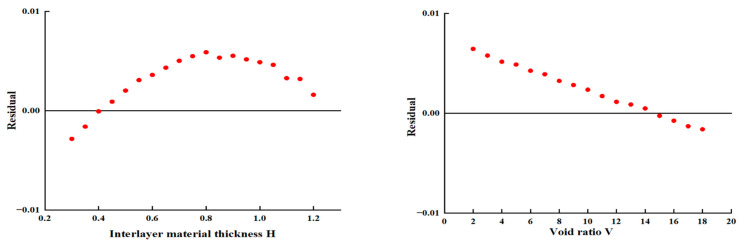

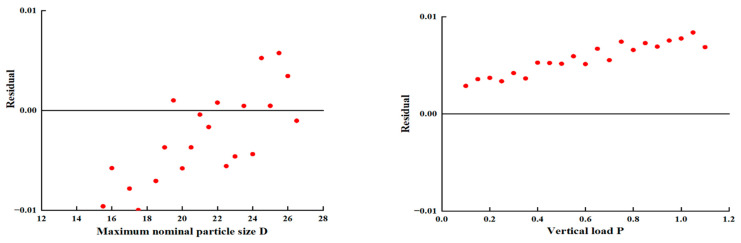
Residual fluctuation plots of each influencing factor.

To further verify the accuracy of Equation (7), this study conducted a residual analysis to evaluate the differences between the simulated values of shear strength τ and the experimental values outside the parameter range searched by the model. As shown in Figure 15, residual distribution plots were generated for four key influencing factors. The results indicate that the residuals of all variables are concentrated around zero with relatively small fluctuations, and there is no obvious systematic deviation or trend. This suggests that the model exhibits strong stability and reliable predictive performance under multivariable conditions. In addition, the residual values remain within a narrow range (±0.01), which further confirms that the model can effectively capture the relationships between influencing factors and interlayer shear strength.

Although the meso-level prediction model demonstrates high accuracy and reliability in fitting performance, its practical engineering application is limited by the difficulty in directly measuring certain mesoscopic parameters—such as void structure or aggregate morphology—using standard field or laboratory techniques. These parameters often lack accessibility and repeatability in routine pavement evaluation processes. To enhance the model’s practicality and facilitate broader application in engineering practice, it is therefore necessary to establish an equivalent prediction model based on measurable macroscopic parameters. By mapping mesoscopic inputs to their corresponding macro-level counterparts, the model can be effectively translated from a theoretical framework into a field-applicable tool, combining predictive accuracy with practical operability. This provides a solid foundation for the development of macro–mesoscale coupled prediction models in future research.

### 5.2. Prediction Model of Macro–Mesoscale Equivalent Interlayer Shear Strength

**Figure 16:**
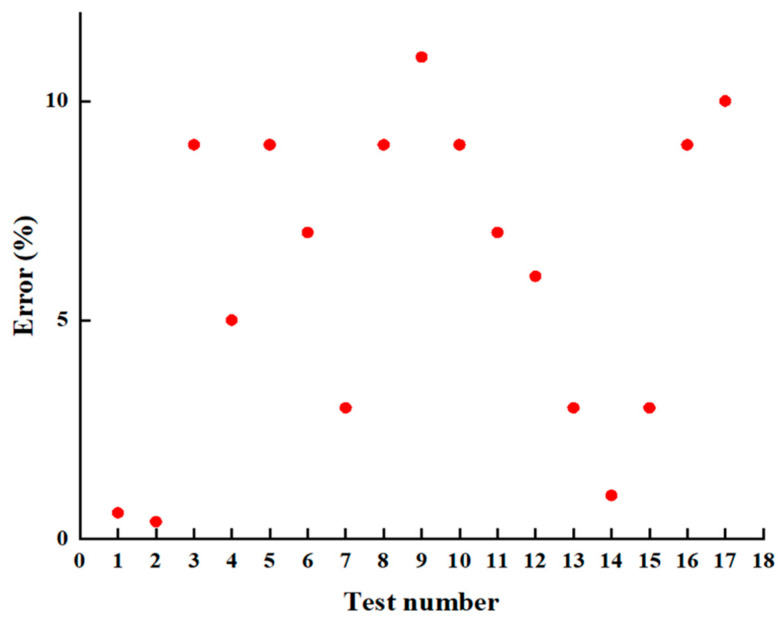
Relative error between the macroscopic tests and mesoscopic simulations.

After obtaining the results of the macroscopic shear tests, they were first compared with the mesoscopic simulation results to evaluate the engineering applicability and accuracy of the simulation model. The relative error R between the macroscopic test values and the mesoscopic simulation values was calculated using the following formula, shown as Equation (8). The positive or negative sign of the error merely reflects the direction of deviation between the simulated values and the actual values. In the analysis, we are more concerned with the actual magnitude of the error. Therefore, to more intuitively measure the accuracy of the model’s predictions, we have taken the absolute value of the error.

(8) $$R=\frac{|SIM-EXP|}{EXP}\times 100\%$$

where R represents the relative error, EXP denotes the macroscopic test value (MPa); and SIM refers to the mesoscopic simulation value (MPa). The relative error between the equivalent macroscopic tests and mesoscopic simulations are presented in Figure 16. Among them, “Test number” refers to the serial number of the test.

The comparative analysis between macroscopic tests and mesoscopic simulations reveals that the relative errors across different influencing factors remain within an acceptable range, confirming the validity of using macroscopic parameters as effective substitutes for mesoscopic variables. The smallest discrepancies—approximately 4%—were observed in the bonding material type and application rate, indicating that the experimental control of the bonding layer distribution was precise and uniform. Notably, the error was minimized at an application rate of 0.9 L/m^2^, underscoring the significance of identifying an optimal bonding quantity. Slightly larger errors, in the range of 7% to 8%, were associated with the maximum nominal aggregate size and void content, likely due to practical challenges in the mixture preparation, including aggregate loss during sieving, uneven mixing, and deviations from the ideal gradation or binder content assumed in the model. For external loading, the relative error was about 5%, which can be attributed to the idealized vertical loading in simulations versus the inevitable dispersion of stress during experimental load transmission. Overall, the alignment between experimental and simulation results validates the mesoscopic model’s accuracy and supports the development of an equivalent macroscopic prediction framework suitable for practical engineering applications.

Subsequently, to accurately evaluate the interlayer shear performance of asphalt pavements with a granular base in practical applications, a prediction model of macro–mesoscale equivalent interlayer shear strength, considering the simulation and experimental results, is established. To further enhance the accuracy and applicability of the macro–mesoscale equivalent interlayer shear strength prediction model, several influencing factors were optimized as follows. First, in terms of air void content, factors such as aggregate loss during sieving, uneven mixing, and compaction errors during specimen preparation may affect the material properties. Therefore, experimental data—which better reflect actual material behavior—are used to replace simulation results for this parameter as well. Second, for external loading, the simulation idealizes load application as purely vertical due to boundary-condition constraints, neglecting the dispersion and lateral stress effects present in real-world experiments. Consequently, the model incorporates experimental data to correct for the influence coefficient of the external load. In contrast, the nominal maximum aggregate size was consistently defined in both experimental and simulation procedures, and mesoscale modeling can more accurately capture the influence of particle size on interlayer shear performance. Thus, this parameter remains based on simulation results. Similarly, the bonding material application rate showed the smallest relative error between the macroscopic tests and simulations, indicating precise and uniform control in the experiments. This also confirms the functional relationship between application rate and interlayer thickness in the mesoscale model, supporting the continued use of simulation data for this factor.

In summary, by combining the strengths of both experimental measurements and simulation results, an optimized prediction model of macro–mesoscale equivalent interlayer shear strength is proposed, in which the most reliable parameters are substituted into the original function. The final model is expressed in Equation (9).

(9) $$\tau =0.084-0.111H+0.144H^{2}-0.06H^{3}+0.02V+0.017D-0.001D^{2}+0.025P-\;0.059P^{2}+0.041P^{3},\;R^{2}=0.984$$

where H is the application rate (L/m^2^); V is the air void content (%); D is the nominal maximum aggregate size (mm); and P is the external load (MPa).

## 6. Conclusions and Outlook

This study developed a macro–mesoscale evaluation framework to assess and predict the interlayer shear performance of asphalt pavement structures incorporating granular base layers. By coupling microscale finite element modeling with macro-scale shear testing, the complex interlocking mechanisms between aggregates at the interlayer interface were effectively characterized and quantified. The key findings are summarized as follows:

(1) SBS-modified 70# asphalt exhibited the highest interlayer bonding performance, with an optimal spreading rate of 0.9 L/m^2^. Among the tested bonding agents, the interlayer shear strength followed this descending order: SBS-modified asphalt > SBR-emulsified asphalt > base bitumen. For all materials, shear strength increased with application rate up to a peak value, beyond which it declined due to excess binder weakening the mechanical interlock.
(2) Increased air void content and larger nominal maximum aggregate sizes in the asphalt mixture both led to reduced interlayer shear strength, highlighting the importance of mixture compaction and gradation design. External loading progressively enhanced shear strength until structural failure occurred, underscoring the necessity of regulating traffic loads to prevent overstressing the interlayer.
(3) A macro–mesoscale interlayer shear-strength prediction model was developed by integrating finite element simulations with equivalent macro-scale experimental parameters. Key influencing factors—including bonding material thickness, air void content, aggregate size, and external loading—were incorporated to enable accurate prediction based on practical design inputs. The model exhibited strong agreement with experimental data and holds significant potential for applications in pavement design, construction quality assessment, and performance-based specifications.

In conclusion, this research provides a robust scientific foundation and practical methodology for evaluating asphalt pavement interlayer performance. Future work should extend the modeling framework to fully three-dimensional simulations, incorporate environmental factors such as moisture infiltration and freeze–thaw cycling, and explore fatigue performance under repeated loading to support life-cycle-oriented pavement design and durability enhancement.

## Figures and Tables

**Table 1:** Prony series parameters.

Materials	${\bar{g}}_{i}$	${\bar{k}}_{i}$	$\tau_{i}$
AC-13	0.4	0.4	1
AC-16	0.4	0.5	1
AC-20	0.2	0.8	0.8
AC-25	0.2	0.8	0.8

**Table 2:** Cohesive-zone model parameters.

Material Type	Interfacial Stiffness (N/mm)	Initial Maximum Shear Stress (MPa)	Maximum Normal Stress (MPa)	Fracture Energy (J/mm)
SBS asphalt	2.65	0.046	0.049	257
SBR asphalt	2.00	0.038	0.038	200
Base bitumen	1.20	0.025	0.027	120

**Table 3:** Material parameters.

Material Type	Thermal Conductivity (W/m·K)	Specific Heat Capacity (J/kg·K)	Thermal Expansion Coefficient (α/K)	Density (g/cm^3^)	Elastic Modulus (MPa)	Poisson’s Ratio
AC-13	1.2	1.1 × 10^−6^	1.8 × 10^−4^	2.35	2500	0.25
AC-16	1.3	1.05 × 10^−6^	1.7 × 10^−4^	2.40	2600	0.25
AC-20	1.4	1.0 × 10^−6^	1.6 × 10^−4^	2.45	2700	0.25
AC-25	1.5	0.95 × 10^−6^	1.5 × 10^−4^	2.50	2800	0.25
Coarse aggregate	2.6	9.2 × 10^−7^	8.7 × 10^−6^	2.4	350	0.35
Fine aggregate	2.6	9.2 × 10^−7^	6 × 10^−6^	2.1	200	0.3
SBS asphalt	0.7	1.65 × 10^−6^	6.1 × 10^−4^	_	_	_
SBR asphalt	0.2	1.9 × 10^−6^	4.5 × 10^−4^	_	_	_
Base bitumen	0.18	1.8 × 10^−6^	4.0 × 10^−4^	_	_	_

**Table 4:** Quantity of crushed stone aggregate in the granular base layer.

Size	31.5	26.5	19	16	13.2	9.5	4.75
Number	0	1	4	4	5	10	43

**Table 5:** Gradations of asphalt mixture surface courses used in this study.

Sieve Size (mm)		26.5	19	16	13.2	9.5	4.75	2.36	1.18	0.6	0.3	0.15	0.075
AC-13	Upper limit	/	/	100	100	85	68	50	38	28	20	15	8
	Lower limit	/	/	100	90	68	38	24	15	10	7	5	4
	Medium value	/	/	100	95	76.5	53	37	26.5	19	13.5	10	6
	Design gradation	/	/	100	92.4	78.6	49	35.9	30	24.1	12.4	7.4	4.7
AC-16	Upper limit	/	100	100	92	80	62	48	36	26	18	14	8
	Lower limit	/	100	90	76	60	34	20	13	9	7	5	4
	Medium value	/	100	95	84	70	48	34	24.5	17.5	12.5	9.5	6
	Design gradation ①	/	100	94.3	82.2	72.6	52.7	40.5	29.6	10.2	13.5	11.3	7
	Design gradation ②	/	100	95.5	84.4	73.1	47.3	32.3	24.3	15.8	10.4	8.9	6
AC-20	Upper limit	100	100	92	80	72	56	44	33	24	17	13	7
	Lower limit	100	90	78	62	50	26	16	12	8	5	4	3
	Medium value	100	95	85	71	61	41	30	22.5	16	11	8.5	5
	Design gradation	100	91.8	88	75.6	65.6	47.9	28.4	17.8	12.5	8.5	7.1	6.2
AC-25	Upper limit	100	90	83	76	65	52	42	33	24	17	14	7
	Lower limit	90	75	65	57	45	24	16	12	8	5	4	3
	Medium value	95	82.5	74	66.5	55	38	29	22.5	16	11	9	5
	Design gradation	94	80.6	76.1	61.4	52.2	35.3	24.4	17.5	12.8	8.3	7.8	6.1
AM-16	Upper limit	100	100	85	68	40	25	18	14	10	8	5	100
	Lower limit	100	90	60	45	18	6	3	1	0	0	0	100
	Medium value	100	95	72.5	56.5	29	15.5	10.5	7.5	5	4	2.5	100
	Design gradation	100	95.4	71.2	50.9	30.2	20.4	15.7	10.8	7.2	5.9	4.3	100
OGFC-16	Upper limit	100	100	90	70	30	22	18	15	12	8	6	100
	Lower limit	100	90	70	45	12	10	6	4	3	3	2	100
	Medium value	100	95	80	57.5	21	16	12	9.5	7.5	5.5	4	100
	Design gradation	100	93.2	85.3	58	19	18.2	11.4	9.6	6	5	3.6	100

In Table 5, “Design gradation ①” and “Design gradation ②” of the AC-16 asphalt mixture represent two different design gradation schemes for this type of mixture, respectively.

**Table 6:** Technical indicators of bonding materials used in this study.

Basic Performance	Penetration (25 °C, 100 g, 5 s, 0.1 mm)	Softening Point (°C)	Ductility (10 °C, 5 cm/min) cm
SBS asphalt	66.2	80.4	120
SBR asphalt	85.3	62.6	120
Base bitumen	92.1	47.6	90

**Table 7:** Gradation composition of the granular base.

Sieve Size (mm)	Aggregate Types (mm)				Composite Gradation	Standard Gradation		
	0–5	5–10	10–20	20–31.5		Lower Limit	Upper Limit	Median Value
31.5	100	100	100	100	100	100	100	100
26.5	100	100	100	85	97.3	90	100	95
19	100	100	72	16	81.2	68	83	75.5
16	100	100	46	4.8	68.8	55	70	62.5
13.2	100	100	16.8	1.7	60.2	48	64	56
9.5	100	69.3	5.2	0.7	46.9	40	52	46
4.75	88.4	8.4	0.6	0.4	26.1	27	38	32.5
2.36	67.2	3.2	0.5	0.2	18.3	17	28	22.5
1.18	43.3	0.7	0.5	0.2	11.1	12	22	17
0.6	36.5	0.6	0.4	0.2	9.5	8	16	12
0.3	20.8	0.5	0.4	0.2	5.8	5	13	9
0.15	17.3	0.5	0.3	0.1	4.1	3	9	6
0.075	7.2	0.5	0.3	0.1	2.1	0	4	2
Ratio	25	30	30	15	100	/	/	/

**Table 8:** Fitted equations for different influencing factors.

Influencing Factors	Simultaneous Equations	R^2^
Interlayer material thickness	y = 0.125 + 0.013 x − 0.009 x^2^	0.893
Void ratio	y = 0.130 − 0.000095 x	0.958
Maximum nominal particle size	y = 0.1298 + 0.00003 x − 0.00007 x^2^	0.927
Vertical load	y = 0.125 + 0.007 x	0.939

## Data Availability

The original contributions presented in this study are included in the article. Further inquiries can be directed to the corresponding author.

## Abbreviations

All abbreviations used in the article are listed below with their full names.

**Abbreviation**	**Full Name**
SBS	Styrene–Butadiene–Styrene
SBR	Styrene–Butadiene Rubber
RAP	Reclaimed Asphalt Pavement
ESAL	Equivalent Single-Axle Load
